# Gaps in Vitamin D Intake and Status in Moroccan Women

**DOI:** 10.3390/epidemiologia6040066

**Published:** 2025-10-17

**Authors:** Noura Zouine, Ilham Lhilali, Abdelhai Messaoudi, Samir El Jaafari, Younes Filali-Zegzouti

**Affiliations:** 1 FSTE-FSM Joint Laboratory “Natural Resources, Health, and Environment”, Faculty of Sciences, Moulay Ismail University, Meknes 50000, Morocco; i.lhilali@edu.umi.ac.ma (I.L.); s.eljaafari@umi.ac.ma (S.E.J.); y.filalizegzouti@fste.umi.ac.ma (Y.F.-Z.); 2 Higher Institute of Nursing and Health Professions of Fes-Meknes Annex, Meknes 50000, Morocco; 3 LREMMALIF—Laboratory for Research in Management, International Logistics Marketing, and Finance, Sidi Mohamed Ben Abdellah University—EST, Fez 30000, Morocco; abdelhai.messaoudi@usmba.ac.ma

**Keywords:** dietary intake, food sources, vitamin D deficiency, 25-hydroxyvitamin D, premenopausal women, Morocco

## Abstract

Background: Vitamin D is essential for women’s health, yet deficiency is widespread among Moroccan premenopausal women. Objectives: This study examined vitamin D intake, dietary sources, determinants, and predictors of serum 25-hydroxyvitamin D [25(OH)D_3_] in 355 women aged 18–49 years in Meknes, Morocco. Methods: Intake and sun exposure were assessed with validated questionnaires, and serum 25(OH)D_3_ was measured by chemiluminescence immunoassay. Multivariable and penalized regression (LASSO) were applied to deseasonalized values. Results: Median intake was 2.89 µg/day, and fewer than 20% of participants met the 5 µg/day recommendation. Fish (48%), dairy (24.39%), and meat (9.40%) were the main sources. Intake varied by age and residence: women aged 18–25 had significantly lower intakes (*p* = 0.027), while rural women consumed less than urban women (2.73 vs. 3.18 µg/day, *p* = 0.014), with inadequacy in 67.70% vs. 32.30% (*p* = 0.018). In adjusted regression, quartiles Q2–Q4 (1.76–16.60 µg/day) were associated with ~+3 ng/mL higher serum 25(OH)D compared to Q1 (0.20–1.76 µg/day, *p* < 0.05). Increments plateaued beyond Q2, and deficiency (<20 ng/mL) persisted in all quartiles (>59%, including 64% in Q4), reflecting limited sun exposure and high adiposity. Sun exposure was a strong positive predictor (β = 0.35, *p* < 0.001), while BMI was inversely associated (β = −0.37, *p* < 0.001). In LASSO, only sun exposure remained, explaining ~3% of variance. Conclusion: In this population, improving sun exposure (≥20 min/day) should be prioritized, alongside increasing vitamin D intake through richer food sources and fortification, while also addressing obesity, with a focus on women at risk of deficiency.

## 1. Introduction

Vitamin D comprises fat-soluble steroid compounds from various sources that share similar structures and biological functions [1]. Often referred to as pro-hormone with complex endocrine regulation [2], it plays a major role in controlling calcium and phosphate homeostasis, normal bone growth, and mineralization [3,4]. It is also crucial in the immune response, promoting an anti-inflammatory state and maintaining the balance between pro- and anti-inflammatory activities [5]. Inadequate vitamin D status is linked to a range of health issues. In children, it can lead to growth retardation and rickets [4,6], while in adults, it is linked to osteoporosis [4], chronic conditions such cardiovascular diseases [7] and cancers [8]. Vitamin D deficiency (VDD) is also associated with adverse pregnancy outcomes and complications in reproductive age such iron deficiency anemia [9], polycystic ovary syndrome (PCOS), endometriosis, and spontaneous miscarriage [10]. These health issues significantly impact quality of life, increase mortality rates, and raise healthcare costs [11,12].

Sunlight remains the primary source of vitamin D globally, with human skin uniquely able to synthesize it endogenously [13]. In response to ultraviolet rays (290–315 nm), 7-dehydrocholesterol in the skin converts to pre-vitamin D3, which then isomerizes to vitamin D3 within the plasma membrane [13]. However, skin synthesis of vitamin D is unreliable due to factors like seasonal variation, high latitude, pollution, and skin pigmentation [14].

VDD has become a global health concern, even in sunny regions [15,16]. In the Mediterranean, Middle East, and North Africa (MENA) region, up to 96% of children, teenagers, and adult women present hypovitaminosis D (25-hydroxyvitamin D [25(OH)D] < 20 ng/mL), with female sex as a significant risk factor [17]. Women’s vulnerability to VDD is linked to physiological factors (pregnancy, lactation, menopause) [18], limited sun exposure due to lifestyle and cultural practices (e.g., sunscreen use, indoor living, covered clothing) [13,17,19], and inadequate vitamin D intake [20].

Vitamin D3 (cholecalciferol) and vitamin D2 (ergocalciferol) are the predominant forms of vitamin D in foods and are effective in maintaining vitamin D status [21,22]. Vitamin D3 (cholecalciferol) is primarily found in animal sources such as fatty fish, fish oil, egg yolks, and red meat, while small amounts of vitamin D2 (ergocalciferol) is synthesized by fungi [23]. Achieving optimal plasma vitamin D levels through diet alone is challenging Food fortification is an effective strategy to increase vitamin D intake, with products like milk, yogurt, juices, and cereals commonly fortified [24]. Likewise, dietary supplements contribute 6% to 47% of total vitamin D intake in some countries [25].

In Morocco, 78.8% of women of reproductive age (WRA) are vitamin D-deficient 25(OH)D < 20 ng/mL), with over one-third (31.3%) severely deficient (25(OH)D < 10 ng/mL) according to the latest national nutritional survey [26]. Despite food fortification efforts 24 years ago [27], the actual dietary intake of vitamin D remains under-documented in the general population. Research on vitamin D status in Moroccan women has mainly focused on sun exposure and lifestyle behaviors as key determinants [28,29,30]. However, a study by Benhammou et al. (2016) found that average vitamin D intake among 101 Moroccan women was only 12.36% of the recommended daily allowance, reflecting the potential adverse impact of Westernized dietary patterns on nutrient intake [31].

Therefore, our study aimed to explore dietary vitamin D intake among Moroccan WRA, identify its determinants, and examine its association with vitamin D status. Addressing dietary behavior may provide a modifiable avenue for improving vitamin D levels in this population.

## 2. Materials and Methods

### 2.1. Study Population and Timeline

This study was conducted in Meknes, north-central Morocco, (latitude 33°52′22.86″ N, sea level), covering all seasons of 2022: spring (March–May), summer (June–August), autumn (September–November), and winter (December–February). Participants were recruited from private medical clinics and laboratories collaborating in the project. The sample size was based on Cochran’s formula [32], using a national VDD prevalence estimate of 78.8% among women of reproductive age. Although the target was 256 women, 533 were recruited to increase power, with 355 meeting inclusion criteria and completing the study.

### 2.2. Questionnaires

Dietary intake was assessed using a validated vitamin D food frequency questionnaire (VitD-FFQ) comprising 78 items, including naturally rich and locally available fortified foods [33]. Each item included four to five portion sizes (g, ml, or household measures) and six frequency categories (monthly to daily) to estimate intake over the past month [33]. Total vitamin D intake (µg/day) was calculated using French/USDA food composition tables. For fortified foods, label-declared values were used. Intake adequacy was assessed based on the WHO/FAO recommendation of 5 µg/day for adult women [34].

Sun exposure behaviors were assessed using a validated questionnaire for Moroccan women of reproductive age [35], evaluating factors influencing cutaneous vitamin D synthesis frequency, duration, time of day, exposed body areas, sunscreen use, and clothing. Skin phototype (types I–VI) was used to adjust individual exposure scores (0.25–1 scale). Based on their Sun Exposure Score (SES), participants were categorized as low (<7.5), moderate (7.5–15), adequate (15–30), or high (>30) [33,35].

Physical activity was assessed using the short-form International Physical Activity Questionnaire (IPAQ-SF), which includes seven items on activities from the past week [36]. Participants were classified into three categories: low (<600 MET-min/week), moderate (≥600), and vigorous (≥3000) activity levels [37].

The data collection process for each participant was conducted through a one-on-one interview with a dietitian and nurses who also collected information on socio-demographic characteristics (age, marital status, occupation, geographic location and education level).

### 2.3. Measures

Anthropometric measurements were taken, with participants being minimally clothed and without shoes. Weight was recorded using a calibrated digital scale (SECA^®^; Hamburg Germany) with 0.5 kg precision), and height was measured to the nearest 0.1 cm using a portable stadiometer (SECA 214, Hamburg Germany). BMI was calculated as weight (kg) divided by height (m^2^) and classified as underweight (<18.5), normal (18.5–24.9), overweight (25–29.9), or obese (≥30 kg/m^2^) [38].

Fasting venous blood (10 mL) was collected in the morning by trained nurses after 8–12 h of fasting. Samples were processed the same day. Serum 25(OH)D was measured using an electrochemiluminescence immunoassay (Architect, Abbott Laboratories, Ilinois, IL, USA). Creatinine was analyzed using the Abbott Architect ci4100 COBAS E411 analyzer.

Vitamin D deficiency (VDD) was defined as serum 25(OH)D_3_ < 20 ng/mL; insufficiency as 20–30 ng/mL; and adequacy as >30 ng/mL [39,40].

To account for renal function, estimated glomerular filtration rate (eGFR) was calculated using the MDRD formula [41]. Only women with eGFR > 60 mL/min/1.73 m^2^ were included in the analysis [42].

### 2.4. Ethics

The study was approved by the Biomedical Research Ethics Committee of Moulay Ismail University (Ethics Approval No:1/CERB-UMI/19.). Procedures followed the principles of the Declaration of Helsinki. Prior to participation, all eligible participants were provided with detailed verbal and written information about the study, including its objectives and procedures. Participants were assured of the voluntary nature of their participation, the right to refuse or withdraw from the study at any time without any consequence, and the confidentiality of their responses.

Written informed consent was obtained from all participants before data collection. For participants with limited literacy, the consent form was read aloud in the presence of a literate impartial witness mainly a family member ‘where found”, and a thumbprint was obtained in lieu of a signature.

### 2.5. Statistical Analysis

We tested normality of continuous variables using Kolmogorov–Smirnov and Shapiro–Wilk tests for samples over 50. Since some variables were not normally distributed, we used non-parametric tests. Continuous variables are shown as means (standard deviations) or medians with interquartile ranges, and categorical data as frequencies and percentages.

Before presenting prevalence estimates Serum 25-hydroxyvitamin D [25(OH)D] concentrations were adjusted for seasonal variation using a cosinor regression model with a fixed 12-month period [43,44]. Two trigonometric terms (sine and cosine of month of blood draw) were included in a linear regression to estimate the seasonal component [43]. Deseasonalized values were obtained by subtracting the predicted seasonal component from each observed concentration and centering on the overall mean, providing season-independent estimates for subsequent analyses. Detailed model parameters (mesor, amplitude, acrophase) and regression metrics are reported in Appendix A Table A1.

Associations between continuous characteristics and dietary vitamin D intake were evaluated using Spearman’s rank correlation (ρ) with corresponding *p*-values. For categorical characteristics, differences in intake were tested using the Kruskal–Wallis test, a Mann–Whitney *U* test (for ordinal variables) or the Chi-square test (for nominal variables), as appropriate. Two-sided tests with α = 0.05 were applied. Participants were also grouped according to whether their dietary vitamin D intake was adequate, and subgroup comparisons were performed using the Chi-square test.

We evaluated the functional form of the association between continuous vitamin D intake (µg/day) and deseasonalized serum 25(OH)D using scatterplots and non-parametric tests. Intake was also categorized into quartiles (Q1–Q4) according to its distribution in the study population, with Quartile 1 (lowest intake) serving as the reference category. Deseasonalized serum 25(OH)D concentrations were used as the dependent variable. Multivariable linear regression models were fitted, adjusting for age (continuous), body mass index (BMI, continuous), socioeconomic status (SES, categorical), physical activity (PAC, continuous score), marital status (binary), localization (urban/rural), education level (categorical), and employment status (binary).

To improve model stability and address potential multicollinearity, we additionally applied penalized regression using the least absolute shrinkage and selection operator (LASSO) [45]. The optimal penalty parameter (λ) was chosen by 10-fold cross-validation, retaining both the value minimizing cross-validation error (λmin) and the more parsimonious value within one standard error(λ_1se_). Model performance was evaluated using mean squared error (MSE), root mean squared error (RMSE), and cross-validated *R*^2^, following recommendations in modern statistical learning [46].

Finally, we profiled intake quartiles by reporting median (IQR) serum 25(OH)D_3_ levels and the prevalence of vitamin D deficiency, overweight/obesity, and low-to-moderate SES in each group.

All statistical analyses were conducted using R version 4.4.1 (R Core Team, 2024) within the RStudio environment (RStudio version 2024.04.2, Posit Software). Data management was conducted with the dplyr and tidyr packages, and results were tabulated with broom. Seasonal adjustment of serum 25(OH)D concentrations was carried out using a cosinor regression model (cosinor2 package). Associations between vitamin D intake and deseasonalized 25(OH)D were first explored with linear regression models (stats package), followed by penalized regression using the LASSO method (glmnet and Matrix packages) with 10-fold cross-validation for model selection. Graphical representations, including scatterplots, boxplots, and bar charts, were generated using ggplot2. A *p*-value < 0.05 was considered statistically significant.

## 3. Results

The demographic characteristics of the participants (*n* = 355) are summarized in Table 1. The median age was 29.00 years (IQR: 11.00), with most women being young or middle-aged (25.35% and 55.50%). Overall, 18.03% were illiterate, 63.90% married, 55.80% unemployed, 67.30% urban residents, and 57.80% were overweight or obese.

The mean estimated vitamin D intake from food was 3.63 µg/day (SD: 2.73) and the median intake, was 2.87 µg/day (IQR: 2.76) which is lower than the required vitamin D intake established by the joint WHO/FAO expert of 5 µg/day(200IU/day). Only 19.72% of the participants meet the WHO/FAO recommended intake for vitamin D (≥5 µg/day), while the vast majority (80.28%) have intakes below this threshold (88.28%). Only eight women were taken medical vitamin D supplementation and were excluded from dietary analysis.

The median sun exposure score was 15.92 (IQR: 10.50), with 56.05% of participants reporting sufficient to high SES. Most participants were recruited in summer (40.28%), followed by autumn (24.22%), spring (18.60%), and winter (16.90%).

**Figure A1:**
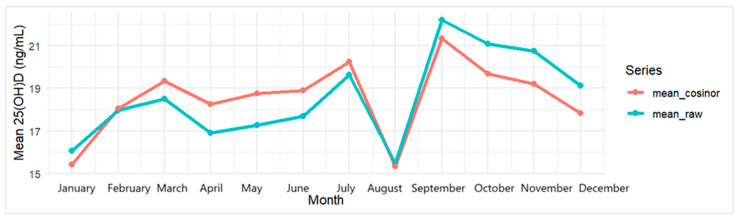
Monthly mean 25(OH)D3: raw vs. deseasonalized (cosinor) in the study sample (*n* = 355).

The median deseasonalized serum 25(OH)D_3_ concentration was 15.61 ng/mL (IQR: 12.46), with 66.20% of participants classified as deficient (<20 ng/mL). Raw values and monthly comparisons are provided in Appendix A Table A2 and Figure A1.

**Figure 1:**
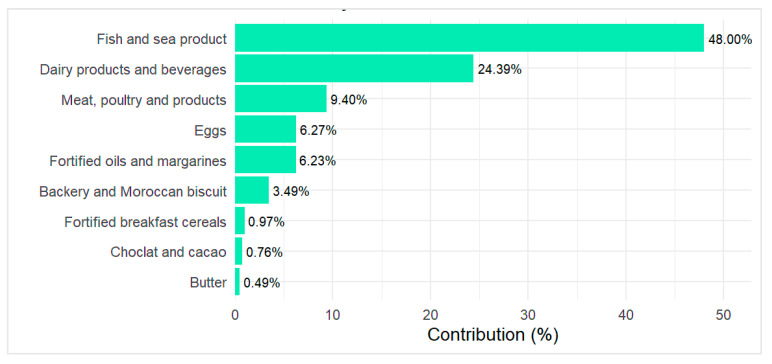
Food sources contribution in the total estimated vitamin D intake.

Figure 1 presents vitamin D food sources among the study participants. The primary food source of the total intake was fish and sea products, contributing to 48%, followed by Milk, dairy products and beverages (24.39%). To a lesser extent, Meat, poultry and related products provide 9.40% while fortified oils and margarines and eggs consumption accounting for 6.27% and 6.23%, respectively, of the total intake. Bakery and Moroccan sweet mostly prepared with eggs and fortified oils account for 3.49% while other commercial fortified foods such as breakfast cereal and cacao provide less than 2% of the intake.

Table 2 illustrate the frequency of consumption for various vitamin D–rich foods by source. Among fish types, fatty fish like sardines (fresh/frozen or canned in tomato sauce) were the most consumed, with over half of participants eating them 1–3 times per month (54.20% and 50.98%, respectively). A smaller proportion reported weekly intake (27.04% and 6.19%). Other fish such as mackerel, white fish, canned tuna/mackerel, and shellfish—were much less frequently consumed, with most participants either abstaining or consuming them less than once per month (ranging from 85.35% for white fish to 91.54% for shellfish).

Meat products rich in vitamin D, like liver, were also rarely consumed, with 81.14% of women reporting low intake. In contrast, poultry (particularly chicken and turkey) was more commonly consumed, with nearly half (45.43%) eating it once a week. Red meat consumption was more limited; 43.66% reported never or rarely consuming it, while only 14.36% reported weekly intake.

Cow’s milk, including fortified varieties, is consumed daily by 40% of participants. Portion-melted cheese is frequently consumed, with 29.28% reporting intake 5–6 times per week and 18.62% daily. Flavored or fruit yogurt is less common, mostly consumed once a week (27.04%). Bakery products are typically eaten weekly by 41.70%. Fortified oil is used daily by 77.18% of participants, though 22.82% report not consuming it. Nearly half (45.63%) do not consume fortified margarine, while 21.15% use it daily. Fortified foods like breakfast cereals, chocolate, and cacao are rarely or never consumed by most participants.

Table 3 presents the correlation between dietary vitamin D intake and studies of demographic factors. A statistically significant relationship was found between vitamin D consumption and age (*p* = 0.002). Notably, young women (less than 36 years) were found to have lowest vitamin D intake than the older one (*p* < 0.027).

Regarding education level, vitamin D intake showed slight variations, although these differences did not reach statistical significance (*p* = 0.351). Similar observations were made regarding the association between vitamin D intake and marital status and employment.

Furthermore, rural women had significantly lower vitamin D intake than their urban counterparts, with median intakes of 2.73 µg/day and 3.18 µg/day, respectively (*p* = 0.014). In addition, 67.70% of rural participants reported inadequate intake, whereas this was observed in only 32.30% of urban participants (*p* = 0.018).

There was no difference in the distribution of vitamin D intake categories across other participant demographic determinants.

**Figure 2:**
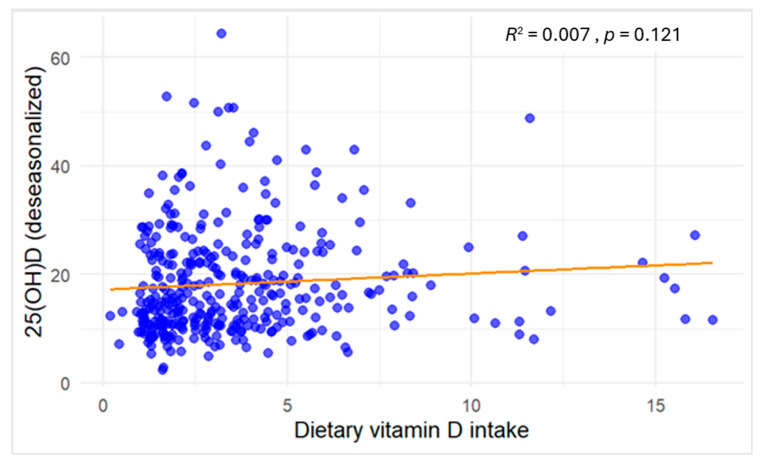
Scatter plot of the association between dietary vitamin D intake (µg/day, continuous) and serum 25(OH)D concentrations (ng/mL) among study participants. Blue dots denote individual participants’ observations, and the orange line depicts the fitted linear regression.

As shown in Figure 2, dietary vitamin D intake demonstrated a negligible association with serum 25(OH)D concentrations (*R*^2^ = 0.007), and the relationship was not statistically significant (*p* = 0.121).

**Figure 3:**
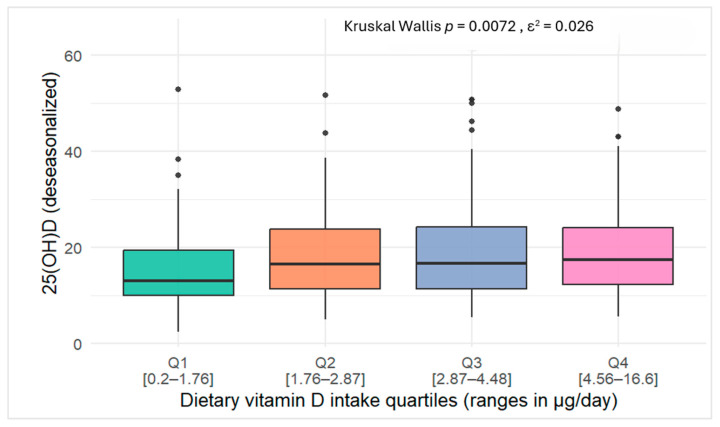
Box plots of serum 25(OH)D concentrations across quartiles of vitamin D intake among study participants.

In Figure 3, serum 25(OH)D concentrations increased progressively across quartiles of dietary vitamin D intake, with significant differences observed between groups (Kruskal–Wallis test, *p* = 0.0076). However, the effect size was small (ε^2^ = 0.026).

In multivariable linear regression analysis (Table 4), higher dietary vitamin D intake was significantly associated with increased circulating 25(OH)D_3_ concentrations. Compared to the lowest quartile (Q1), β (SE) values were 3.33 (0.38), *p* = 0.017 for Q2; 3.24 (1.39), *p* = 0.021 for Q3; and 3.17 (1.39), *p* = 0.023 for Q4. Although significant across quartiles, the effect did not follow a clear dose–response pattern.

BMI was inversely associated with serum 25(OH)D concentrations (β (SE): −0.37 (0.10), 95% CI: −0.57 to −0.17, *p* < 0.001), indicating lower vitamin D levels with increasing BMI. In contrast, SES showed a positive association (β (SE): 0.35 (0.06), 95% CI: 0.22 to 0.47, *p* < 0.001), with higher scores linked to increased 25(OH)D concentrations.

Moreover, serum vitamin D levels were not influenced by sociodemographic characteristics. The overall explanatory power of the model remained modest, accounting for approximately 15.6% of the variance in deseasonalized 25(OH)D (*R*^2^ = 0.156, adjusted *R*^2^ = 0.129, *F*(11, 343) = 5.75, *p* < 0.001).

In penalized regression (LASSO with 10-fold cross-validation) (Table 5), the minimum-error solution (λ.min = 0.079) achieved a CV-MSE of 86.28 (CV-RMSE = 9.29, CV-*R*^2^ = 0.108). Under the more parsimonious λ.1se criterion (λ.1se = 2.24), CV-MSE (93.7) and CV-RMSE (9.68) increased slightly, while CV-*R*^2^ dropped markedly to 0.030. At this level of penalization, only SES was retained in the model, explaining ~3% of the variance in deseasonalized 25(OH)D levels, whereas quartiles of vitamin D intake and other covariates were shrunk to zero.

Table 6 presents the descriptive statistics of serum 25(OH)D concentrations and selected lifestyle characteristics across quartiles of dietary vitamin D intake. Median serum 25(OH)D_3_ concentrations increased gradually with higher intake, from 13.02 ng/mL in the first quartile to 17.35 ng/mL in the fourth quartile. Nevertheless, deficiency remained prevalent in all groups (>59%), including the highest intake quartile (Q4: 4.46–16.60 µg/day), where 64.00% of participants were still classified as deficient. The proportion of participants with high BMI remained consistently above 58% across quartiles, while low/moderate SES was observed in approximately 40–47% of participants.

## 4. Discussion

This study examined dietary vitamin D intake, food sources, and serum 25(OH)D status among Moroccan premenopausal women. Reported intake levels were below recommended values, particularly among younger and rural participants, and fortified foods or other rich sources were rarely consumed. Consistent with these findings, a high prevalence of vitamin D deficiency was observed. In multivariable regression analyses, serum 25(OH)D concentrations were associated mainly with BMI and sun exposure rather than diet. However, in penalized regression (LASSO), only SES was retained, indicating that its effect was more stable under penalization, whereas dietary intake, BMI, and other predictors were shrunk to zero. These findings summarize the main patterns observed for intake and vitamin D status in this population, with each determinant considered in detail in the following sections:

Average intake

The average vitamin D intake from food was 3.63 (2.73) µg/day, which is in line to the mean intake reported in women from countries with comparable dietary patterns and cultural practices, such as Saudi Arabia (3.52 µg/day) [47], Libya (3.9 µg/day) [48], and Egypt (3.27 ± 3.53 µg/day) [49].

Nationally, Benhamou et al. [31] reported a much lower vitamin D intake of 1.85 µg/day in 101 Moroccan women compared to our participants. Yet, accurate comparison of vitamin D intake between studies is challenging due to variations in dietary assessment methodologies [20,50]. We used a vitamin D–specific, validated FFQ for Moroccan WRA over one month, while their study used a Mediterranean diet–focused FFQ adapted from other regions over a 12-month recall potentially leading to underestimation of daily vitamin D intake due to recall bias. Differences in age groups and food composition tables further limit comparability.

Adequacy of intake and food sources

International guidelines on adequate vitamin D intake remain controversial. The IOM, EFSA, and Endocrine Society recommend 15 µg/day (600 IU) to reach 20 ng/mL in adults with minimal sun exposure. Nordic countries, France, and the UK suggest 10 µg/day, while DACH countries recommend up to 20 µg/day [51]. The WHO/FAO advises 5 µg/day for individuals aged 0–50, including pregnant and lactating women, assuming sufficient UVB exposure [34]. Morocco adopts the same reference in its national fortification policy [52].

Our findings indicate that 88.28% of participants did not meet an adequate intake of 5 μg/day. When compared to the Institute of Medicine’s recommendation of 15 μg/day, only 19.33% of women achieved sufficient intake. Similar inadequacies have been reported across regions, including the Middle East [47,53], North Africa [48,54], the US [55], and Canada [56]. In contrast, Finland’s 2012 FINDIET survey reported an average intake of 8.6 μg/day, sufficient for more than 86% of women, a result attributed to the mandatory fortification of dairy and fat products introduced in 2010 [57].

In Morocco, only a few staple foods are voluntarily fortified with vitamin D. About 90% of edible oils and most margarines contain 7.5 μg/100 g of vitamin D3, covering roughly 30% of daily needs [52,58]. Pasteurized cow milk was formerly fortified at 0.85 μg/100 mL, meeting 17% of daily requirements, but most commercial milk is now half-skimmed and lacks clear fortification labeling, except for some ultra-high-temperature and lactose-reduced products. Certain plant-based milks, yogurts, and cereals indicate vitamin D content exceeding 0.95 μg/100 mL.

Nevertheless, achieving adequate intake through fortification alone remains difficult. To meet 5 μg/day, one would need to consume 4–5 tablespoons of margarine, 6 of oil, or 600 mL of fortified milk daily, an intake unlikely to be maintained consistently across the population.

Our analysis of vitamin D food sources confirmed that fortified products were rarely consumed daily. While 77.18% of participants reported daily use of edible oil, 46% and 88% did not consume margarine or breakfast cereals, respectively. As a result, these foods contributed only 6.19% to total vitamin D intake. Dairy products, including fortified milk, were consumed daily by 58.6% of women and provided 24.5% of total intake. However, the widespread consumption of tea (97.29%) may reduce dairy beverage intake, limiting the potential benefits of vitamin D fortification on 25(OH)D status [26].

Major dietary sources of vitamin D vary across countries depending on fortification policies. In the United States, where fortification is mandatory, milk contributes up to 39% of total intake among women. In Canada, various fortified milk products, including soy and powdered milk, account for 27.1%, with margarine and meat or alternatives contributing 17.3% and 17.8%, respectively [56]. In Finland (FINDIET study), fortified foods provide 38% of total intake, primarily from dairy and fat spreads, while fish contributes 18% [57]. According to the French national INCA 3 study, intake stems mainly from meat, fish, eggs, and derived products (39%), followed by dairy (25%) [59].

In our study, fish was the primary dietary contributor to vitamin D intake (48%), followed by meat and poultry (9.4%) and eggs (6.27%). This profile reflects a Mediterranean dietary pattern characterized by moderate fish and low meat consumption. Comparable trends have been reported in Libya [48], Egypt [49], and Spain [60], where fish accounts for 63%, 56.26%, and 30.4% of women’s intake, respectively. Sardines, whether fresh or canned, were the most frequently consumed, followed by mackerel. As the world’s largest producer and exporter of sardines [61], Morocco provides an accessible source of vitamin D3 (14 µg/100 g fresh, 12.5 µg/100 g grilled). Promoting regular consumption of sardines (1–2 servings/week), using appropriate preparation methods, could improve vitamin D status among premenopausal Moroccan women [62], with potential benefits for bone health [63], breast cancer [64], and metabolic syndrome [65].

Vitamin D dietary intake determinants

Young and rural women should be prioritized when addressing determinants of vitamin D intake. In our sample, women aged 18–25 had significantly lower intake and lower achievement of the recommended intake. Similar findings were reported by Brouzes et al. (2020) among 130 Egyptian women aged 19–30, where 82% had inadequate intake despite high energy and sodium consumption [49]. Zareef et al. (2018) also found age to be a significant determinant of adequate intake in 257 Saudi women, with higher intake among those aged 31–50 (44%) compared to 20–30-year-olds (25%) [47]. In Morocco, systematic vitamin D supplementation remains uncommon outside of medical prescriptions [66]. In our analysis, we could not conclude the impact of supplement use on total intake due to limited information on the types of supplements consumed and the low number of supplement users, who were excluded.

Other sociodemographic factors have been associated with vitamin D intake. While prior studies show that higher education is linked to healthier diets and better adherence to vitamin D recommendations [67,68,69,70,71], we found no significant association. Women without formal education had slightly lower intakes, but the difference was not statistically significant, possibly due to their low representation (18%).

Urban residence was significantly associated with higher vitamin D intake and a greater proportion of participants meeting the recommended intake. Similar findings in Lebanon reported lower intake in rural women and greater consumption of fortified foods among urban residents. In Morocco, rural women often face limited access to fresh, nutrient-rich foods due to geographic and market constraints [26,70]. The COVID-19 crisis has worsened poverty and food insecurity in rural households, further reducing intake of animal-source vitamin D foods [72].

Predictors of serum 25(OH)D

The mean serum 25(OH)D_3_ concentration was 15.64 ng/mL (±12.4), with only 11.8% of participants reaching optimal levels. Vitamin D inadequacy was therefore highly prevalent, consistent with previous reports in Moroccan women and other populations in the region [26,73].

In multivariable regression, higher BMI was inversely associated with serum 25(OH)D_3_ (β = −0.37, *p* < 0.001), consistent with the established role of adiposity in sequestering vitamin D and reducing its bioavailability [74]. Overweight and obesity were common in this sample, affecting more than 58% of participants, which likely contributed to the persistence of deficiency.

The sun exposure score (SES), derived from a validated questionnaire, was a strong positive predictor (β = 0.35, *p* < 0.001) of 25(OH)D_3_ levels, consistent with evidence that sunlight remains the primary determinant of vitamin D status [14]. This is in line with findings from a Moroccan national survey, which reported a lower prevalence of deficiency among women exposed to sunlight (29.6% vs. 36.7%) and particularly among those exposed for more than 20 min per day (27.6%). Nevertheless, in our sample, more than 44% of participants reported insufficient or moderate exposure, reflecting cultural practices such as sun avoidance and the use of protective clothing [18,20]. These patterns help explain why deficiency remained widespread despite adequate dietary intake in some women.

Across different analytical approaches, the contribution of dietary intake to serum 25(OH)D_3_ levels appeared modest. In crude linear models, no association was observed, whereas the Kruskal–Wallis test showed significant differences across intake quartiles (*p* = 0.0076), although the effect size was small (ε^2^ = 0.026). In adjusted regression, women in quartiles 2–4 had ~+3 ng/mL higher concentrations compared to Q1 (*p* < 0.05). These findings confirm an effect of food sources on serum 25(OH)D_3_ levels in the absence of supplementation, consistent with observations from Asakura and colleagues in Japan [75]. In their study of 107 healthy adults at similar latitudes, dietary intake was linearly associated with serum 25(OH)D_3_, with a 1 μg/1000 kcal increase linked to rises of 0.88 ng/mL in summer and 1.7 ng/mL in winter [75]. By contrast, our results showed a plateau beyond Q2, with regression coefficients declining slightly from 3.33 in Q2 to 3.24 in Q3 and 3.17 in Q4, suggesting greater increments observed at lower baseline 25(OH)D levels and diminishing responses at higher levels [76,77]

Mechanistically, this plateau may reflect both biological and contextual factors: reduced absorption efficiency at higher intakes, metabolic self-regulation of vitamin D synthesis, and the modifying role of body composition. In our sample, deficiency persisted across all quartiles (>59%), including Q4 (4.46–16.60 µg/day), where 64% of women remained deficient. Profiling results reinforce this interpretation, as overweight/obesity affected more than 58% of participants and 40–47% reported low-to-moderate sun exposure, both of which likely limited the effectiveness of diet alone in correcting deficiency. Taken together, these results suggest that increasing intake can benefit deficient women, but higher doses even current recommendations on their own are unlikely to resolve widespread deficiency without concurrent improvements in sun exposure and weight status.

In penalized models, neither BMI nor dietary intake persisted, suggesting that their effects were less stable once model complexity was reduced. In contrast, SES was retained, although it explained only ~3% of the variance in deseasonalized 25(OH)D. This weak contribution likely reflects both the modest predictive power of the available variables and the reliance on questionnaire-based estimates rather than objective dosimetry, which in similar latitudes has shown stronger associations with sun exposure [75]. It is also possible that residual confounding contributed to the limited explained variance. Sociodemographic variables and physical activity did not show significant effects in our regression models, yet these factors may interact indirectly with sun exposure or dietary patterns and could be better captured in larger or more heterogeneous samples.

## 5. Limits and Strengths

This study has several strengths and limitations. We used validated, context-specific tools to assess vitamin D intake (FFQ) and sun exposure (SES questionnaire), which increased measurement accuracy. To our knowledge, this is the first study in Morocco to evaluate the combined contributions of dietary and sun-derived vitamin D to serum concentrations. A further strength is the use of deseasonalized 25(OH)D values, which accounted for seasonal variation and allowed inclusion of participants recruited throughout the year. In addition, the collection of detailed covariate data enabled adjustment for multiple potential confounders, strengthening the validity of the findings.

Nevertheless, some limitations should be acknowledged. The sample size was determined using a power calculation focused on vitamin D deficiency among women in Meknes, which may limit generalizability to all premenopausal Moroccan women. Furthermore, energy-adjusted intake analyses were not applied, as the VitD-FFQ was not designed for this purpose; instead, we examined absolute intake in both continuous and categorical forms in relation to sociodemographic characteristics.

## 6. Conclusions

Vitamin D intake remains insufficient among premenopausal Moroccan women, particularly in younger and rural groups. Current voluntary fortification strategies are inadequate to reduce widespread deficiency. Fortifying staples such as wheat flour and extending fortification to dairy products could offer a cost-effective solution. Beyond dietary interventions, improving sun exposure and addressing obesity are also critical. Despite abundant sunlight, cultural and behavioral practices often limit exposure. A combined approach linking nutrition policy, safe sun exposure education, and obesity prevention may provide a sustainable strategy to improve vitamin D status in this population.

## Figures and Tables

**Table 1:** Participants’ general characteristics estimated dietary vitamin D intake (*n* = 355).

Variables	All Participants (*n* = 355)
Age (year). median (IQR)	29.00 (11.00)
Age classe (*n*. %)	
18–25	90 (25.35)
26–36	197 (55.50)
37–49	68 (19.15)
Education (*n*. %)	
Illiterate	64 (18.03)
≤10 years (secondary college)	166 (46.76)
≥11 years (university or higher)	125 (35.21)
Marital status (*n*. %)	
Married	227 (63.90)
Unmarried	128 (36.10)
Employment (*n*. %)	
Yes	157 (44.20)
No	198 (55.80)
Localization (*n*. %)	
Urban	239 (67.30)
Rural	116 (32.70)
BMI (Kg/m^2^), Median (IQR)	25.70 (6.20)
Normal weight (BMI: 18.50–24.99 kg/m^2^)	145 (40.85)
Underweight (BMI < 18.50 kg/m^2^)	5 (1.41)
Overweight (BMI: 25.00–29.99 kg/m^2^)	133 (37.46)
Obese (BMI ≥ 30.00 kg/m^2^)	72 (20.28)
Vitamin D intake (µg/day), Mean (SD)	3.63 (2.73)
Median (IQR)	2.87 (2.76)
Intake adequacy (WHO/FAO RI) (*n*. %)	
Adequate (≥5 µg/day)	70 (19.72)
Low (<5 µg/day)	285 (88.28)
Sun exposure score, Median (IQR)	15.92 (10.50)
Sun exposure categories (*n*. %)	
Insufficient-moderate	156 (43.95)
Sufficient-high	199 (56.05)
Physical activity MET min/week, Median (IQR)	1778.20 (1765.00)
Physical activity level categories (*n*. %)	
Moderate-low	330 (92.96)
High	25 (7.04)
Season (*n*. %)	
Summer	143 (40.28)
Autumn	86 (24.22)
Winter	60 (16.90)
Spring	66 (18.60)
Deseasonalized 25(OH)D (nmol/L), Mean (SD)	18.32 (9.83)
Median (IQR)	15.61 (12.46)
VDD (25(OH)D < 20 ng/mL)	235 (66.20)
VD Insufficiency (20 ≤ 25(OH)D < 30 ng/mL)	80 (22.53)
VD Sufficiency (25(OH)D ≥ 30 ng/mL)	40 (11.27)

Abbreviations; IQR: interquartile range, VDD: vitamin D deficiency, MET: Metabolic Equivalent of Task.

**Table 2:** Percent frequency of consuming vitamin D in foods sources (*n* = 355).

Food Sources	Items	Never or Less Than Once Per Month	1–3 Times Per Month	1 Time Per Week	2–4 Days Per Week	5–6 Days Per Week	Every Day
Fish and sea products	Fatty fish fresh and frozen (Sardin)	18.76	54.20	27.04	0.00	0.00	0.00
	Fatty fish fresh and frozen (Mackerel)	87.32	8.16	4.52	0.00	0.00	0.00
	White fish fresh and frozen (sole, whiting, sea bream…)	85.35	0.00	12.39	2.26	0.00	0.00
	Sardine, canned in tomato sauce	42.83	50.98	6.19	0.00	0.00	0.00
	Tuna and mackerel, canned in vegetable oil	87.32	12.68	0.00	0.00	0.00	0.00
	Shellfish (calmer, scampi, Shrimps, mussels)	91.54	8.46	0.00	0.00	0.00	0.00
Meat products	Red meat (Beef, veal. sheep...), eaten with or without fat	43.66	20.56	14.36	16.61	4.81	0.00
	Organ meats (kidney)	93.53	4.22	2.25	0.00	0.00	0.00
	Organ meats (liver)	81.14	16.61	2.25	0.00	0.00	0.00
	Poultry (chicken and turkey)	14.36	6.19	45.53	22.53	12.38	0.00
	Smoked meats or charcuterie (chorizo, salami, Ham, casheer, mortadella, etc.)	84.79	8.45	3.50	3.26	0.00	0.00
Entire eggs	Eggs (Pan-fried, crambled and boiled)	38.60	18.60	28.16	4.22	10.42	0.00
Dairy Products and beverages	Cow milk vitamin D fortified. Farm-fresh cow’s milk	12.40	0.00	2.25	24.50	20.85	40.00
	Milk drink, or drinking yogurt, flavored or with fruit, sweetened	52.11	25.07	18.58	1.97	2.27	0.00
	Yogurt flavored or with fruit. Sweetened. Non-reduced fat	25.07	22.25	27.04	18.60	7.04	0.00
	Semi-hard cheese (Gouda. Edam. Munster)	81.12	8.16	8.16	2.56	0.00	0.00
	Fresh cheese (ricotta. Mozarelle.)	95.8	4.20	0.00	0.00	0.00	0.00
	Soft cheese (Camembert. Brie)	87.60	4.22	6.47	1.71	0.00	0.00
	Melted cheese in portions or spreadable cubes	0.00	22.81	25.07	4.22	29.28	18.62
	Cottage cheese natural or aromatic	93.80	2.25	2.25	0.00	1.70	0.00
Fat Products	Vegetable oil vitamin D fortified	22.82	0.00	0.00	0.00	0.00	77.18
	Margarine vitamin D fortified	45.63	0.00	4.22	24.78	4.22	21.15
	Butter	42.81	4.22	2.25	22.81	4.22	23. 66
Breakfast cereals	Cereal vitamin D fortified	87.32	6.19	6.49	0.00	0.00	0.00
Chocolate	Chocolate bars (with milk or dark 70% cocoa minimum or with dried fruits)	66.47	14.64	10.42	4.22	4.25	0.00
Cacao	Sweet cocoa or chocolate powder for drinks, enriched with vitamins and minerals	87.32	0.00	8.16	4.52	0.00	0.00
Backery and Moroccan biscuit	Ordinary homemade cake or prepackaged Madeleine. Viennoiserie (krachel, chocolate bread, raisin bread, croissant, brioche) Moroccan biscuits (thee dry biscuit, Fekkas, date biscuit)	18.87	10.42	41.70	6.47	22.54	0.00

**Table 3:** Socio-demographic determinant of vitamin D dietary intake in study participants (*n* = 355).

Characteristics	Dietary Intake (µg/d)						
	Mean (SD)	Median (IQR)	Test Statistics	*p*-Value	Below RI (*n*, %)	Test Statistics	*p*-Value
Age (years)			ρ = 0.162	0.002 **			
Age classes							
18–25	3. 32 (2.58)	2.62 (2.30)	*H* (2) = 7.068	0.029 *	67 (23.50)	*χ*2 (2) = 3.851	0.146
26–36	3.58 (2.73)	2.79 (2.68)			160 (56.10)		
37–49	4.16 (2.84)	3.67 (3.25)		0.027 ^a^	58 (20.40)		
Education							
Illiterate	3.70 (3.39)	2.79 (2.92)	*H* (2) = 2.093	0.351	53 (18.60)	*χ*2 (2) = 4.105	0.128
≤10 years (Secondary-college)	3.86 (2.84)	2.93 (2.11)			125 (43.90)		
≥11 years (University or higher)	3.28 (2.11)	2.87 (2.64)			103 (37.50)		
Marital status							
Married	3.73 (2.85)	2.93 (2.84)	*U* = 15,181.00	0.471	177 (62.1)	*χ*2 (1) = 1.514	0.219
Unmarried	3.44 (2.48)	2.63 (2.73)			108 (37.9)		
Employment							
Yes	3.57 (2.57)	2.82 (2.83)	*U* = 15,486.500	0.982	122 (42.80)	*χ*2 (1) = 0.661	0.414
No	3.67 (2.84)	2.93 (2.75)			163 (57.20)		
Localization							
Rural	3.27 (2.33)	2.73 (2.46)	*U* = 16,793.500	0.014	193 (67.70)	*χ*2 (1) = 5.614	0.018 *
Urban	4.25 (3.22)	3.18 (3.93)			92 (32.30)		

Abbreviations; IQR: interquartile range, RI: recommanded intake. ρ: Spearman Rank correlation coefficient, *H* = Kruskal–Wallis test statistic; ^a^: pairwaiz comparaison, *p* adjusted for Bonferroni correction for multiple test; *U* = A Mann–Whitney test, *χ*2 =: Chi-squar test. * *p* < 0.05, ** *p* < 0.01.

**Table 4:** Key predictors of circulating 25(OH)D levels identified via linear regression analysis.

Predictor	Coefficient (β)	SE	95% CI (LL, UL)	t-Value	*p*-Value
Intercept	15.31	4.49	6.48, 24.14	3.41	<0.001
Vitamin D intake					
Q2 (vs. Q1)	3.33	0.38	0.61, 6.05	2.41	0.017
Q3 (vs. Q1)	3.24	1.39	0.50, 5.97	2.33	0.021
Q4 (highest vs. Q1)	3.17	1.39	0.44, 5.91	2.28	0.023
Age	0.15	0.08	−0.01, 0.29	1.87	0.063
BMI	−0.37	0.10	−0.57, −0.17	−3.53	<0.001
SES	0.35	0.06	0.22, 0.47	5.58	<0.001
PAC	−0.00	0.00	−0.00, 0.00	−1.60	0.110
Localization	0.33	1.06	−1.75, 2.42	0.32	0.752
Education level	0.65	0.72	−0.77, 2.06	0.90	0.372
Employment	0.12	1.00	−1.84, 2.07	0.12	0.907
Marital status	0.53	1.05	−1.55, 2.61	0.50	0.617
*F*(11, 343) = 5.75, *p* < 0.001, *R*^2^ = 0.156, Adjusted *R*^2^ = 0.129.					

Abrreviations, SE: Standard Error, CI: confidence interval; LL = lower limit; UL = upper limit, SES: Sun exposure score, BMI: Body Mass Index.

**Table 5:** LASSO Regression Coefficients After 10-fold Cross-Validation.

Predictor	λ.min (0.079)	λ.1se (2.24)
Intercept	16.27	16.74
Q2 (vs. Q1)	2.76	—
Q3 (vs. Q1)	2.73	—
Q4 (vs. Q1)	2.67	—
Age	0.13	—
BMI	−0.35	—
SES	0.34	0.09
PAC	−0.00	—
Localization	0.06	—
Education level	0.47	—
Employment	0.02	—
Marital status	0.30	—
Models’ performance metrics	CV-MSE = 86.28, CV-RMSE = 9.29, CV-*R*^2^ = 0.108.	CV-MSE = 93.7, CV-RMSE = 9.68, CV-*R*^2^ = 0.030

λ.min = minimum cross-validation error; λ.1se = most parsimonious model within one standard error; “—“ = coefficient shrunk to zero. CV-MSE = cross-validated mean squared error (average squared prediction error across 10-fold cross-validation); CV-RMSE = cross-validated root mean squared error (prediction error in the original measurement units of serum 25(OH)D, ng/mL); CV-*R*^2^ = cross-validated coefficient of determination (proportion of variance in serum 25(OH)D explained by the penalized model under cross-validation).

**Table 6:** Descriptive statistics of serum 25(OH)D by quartiles of dietary vitamin D intake among study participants.

Quartile of Intake	*n*	Median 25(OH)D (ng/mL)	IQR	Deficient (%)	BMI ≥ 25.00 kg/m^2^ (%)	Low/Moderate SES (%)
Q1 (0.20–1.76)	89	13.02	9.50	76.40	58.43	42.70
Q2 (1.76–2.87)	89	16.54	12.33	59.55	59.55	41.57
Q3 (2.87–4.48)	89	16.61	12.88	65.17	60.67	47.19
Q4 (4.56–16.60)	88	17.35	11.89	63.64	60.23	44.32

## Data Availability

The datasets generated and analyzed for the current study are available from the corresponding author upon reasonable request.

## Appendix A

**Table A1:** Cosinor regression results for seasonality of serum 25(OH)D3 in the study sample (*n* = 355).

Metric	Estimate	Std.Error	t-Value	*p*-Value
Intercept (MESOR)	18.338	0.539	33.984	3.405
Cosine coefficient (βcos)	1.247	0.822	1.516	0.130
Sine coefficient (βsin)	−0.867	0.708	−1.225	0.221
Amplitude	1.519	-	-	-
Acrophase (months)	1.160	-	-	-
*R* ^2^	0.009	-	-	-
Adj *R*^2^	0.003	-	-	-
Global seasonality *p*-value	0.185	-	-	-

MESOR = Midline Estimating Statistic Of Rhythm. Cosine and sine coefficients are raw regression terms with associated standard errors, t- and *p*-values. Amplitude and acrophase are derived from these coefficients. Global seasonality *p*-value tests the joint significance of the cosine and sine terms.

**Table A2:** Raw and deseasonalized 25(OH)D3 concentrations and deficiency prevalences among study participants (*n* = 355).

Category	Raw 25(OH)D3	Deseasonalized 25(OH)D3
Mean (SD)	18.32 (9.88)	18.32(9.83)
Median (IQR)	15.70 (12.32)	15.61 (12.46)
Status (*n*. %)		
Deficiency (<20 ng/mL)	231 (65.07)	235 (66.20)
Insufficiency (20–29.9 ng/mL)	84 (23.66)	80 (22.53)
Sufficiency (≥30 ng/mL)	40 (11.27)	40 (11.27)

SD: standard deviation, IQR: interquartile range.
